# Autophagy-related genes genetically interact with Pmk1 MAPK signaling in fission yeast

**DOI:** 10.17912/micropub.biology.000618

**Published:** 2022-08-04

**Authors:** Teruaki Takasaki, Ryosuke Utsumi, Erika Shimada, Naofumi Tomimoto, Ryosuke Satoh, Reiko Sugiura

**Affiliations:** 1 Laboratory of Molecular Pharmacogenomics, Department of Pharmaceutical Sciences, Faculty of Pharmacy, Kindai University, Higashiosaka, Osaka, Japan

## Abstract

Apart from the highly conserved role in the cellular degradation process, autophagy also appears to play a key role in cellular proliferation. Here, we describe the genetic interaction of autophagy-related genes and Pmk1 MAPK signaling in fission yeast. *atg1* deletion cells (Δ *atg1* ) exhibit the *vic* (viable in the presence of immunosuppressant and Cl ^-^ ) phenotype, indicative of Pmk1 signaling inhibition. Moreover, the Δ *atg1* Δ *pmk1* double mutant resembles the single Δ *pmk1* mutant, suggesting that Atg1 functions in the Pmk1 pathway. In addition, the growth defect induced by overexpression of Pck2, an upstream activator of Pmk1 MAPK was alleviated by the deletion of *atg1 ^+^* . Finally, the deletion of autophagy-related genes recapitulates Pmk1 MAPK signaling inhibition. Our data suggest a novel role for autophagy in MAPK signaling regulation.

**Figure 1. Deletion of autophagy-related genes phenotypically recapitulates Pmk1 MAPK signaling inhibition:**
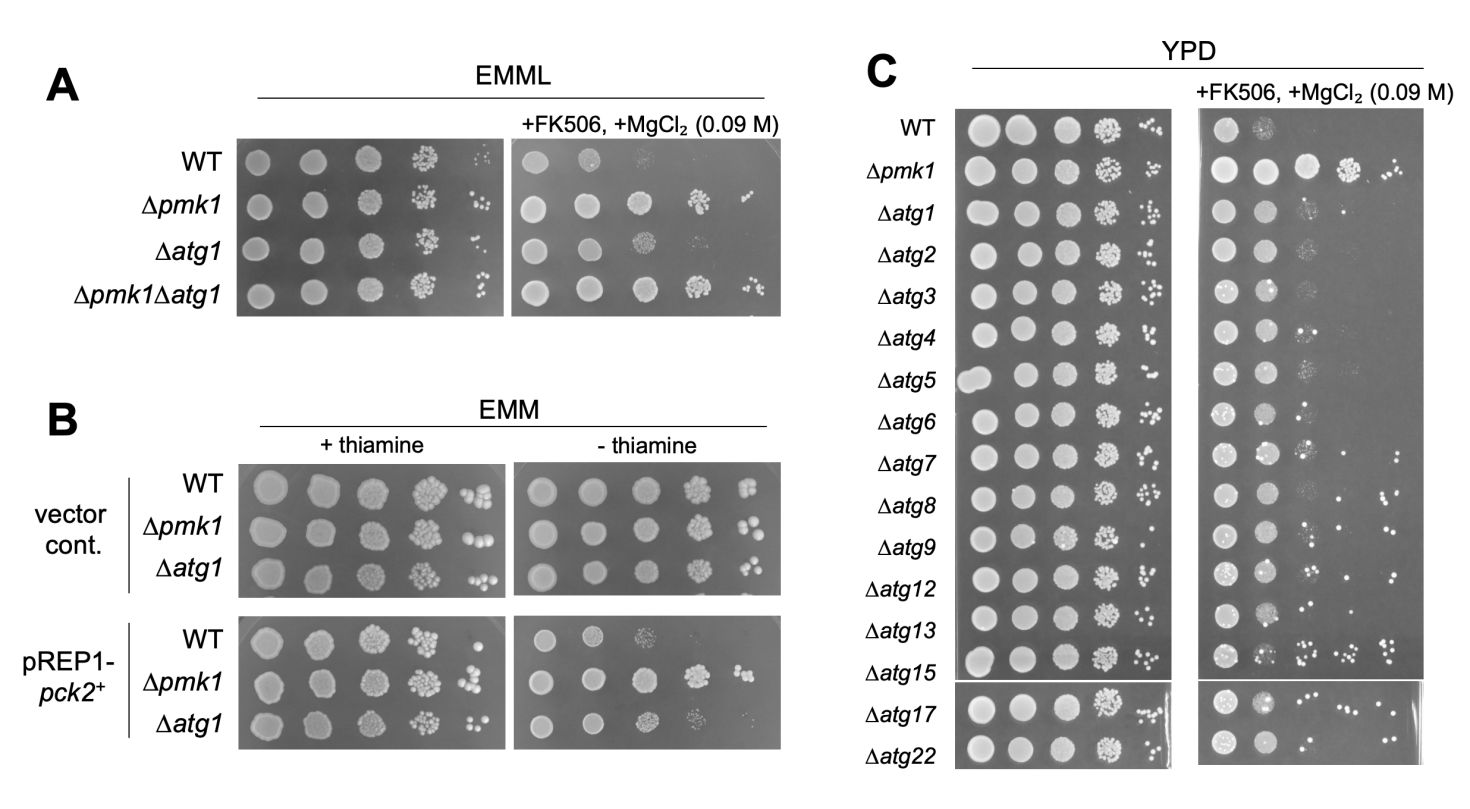
**A:** Strains grown in EMM with leucine (EMML) were serially diluted and spotted onto EMML plates with or without FK506 and 0.09 M MgCl _2_ , then incubated at 27˚C for 4 days. **B:** Strains grown in EMM with thiamine were serially diluted and spotted onto EMM plates with or without thiamine, then incubated at 27˚C for 7 days. **C:** WT, *pmk1* deletion cells (Δ *pmk1* ), and cells deleted for autophagy-related genes were grown in YPD and serially diluted and spotted onto YPD plates with or without FK506 and 0.09 M MgCl _2_ , then incubated at 27˚C for 3 days.

## Description

Autophagy is an evolutionarily highly conserved mechanism to maintain cellular homeostasis via waste clearance using the lysosomal machinery (Mukaiyama *et al.* 2010; Mizushima and Komatsu 2011). Although starvation potently induces autophagy, the basal level of autophagy is maintained even in normal growth conditions, thereby controlling development, cellular metabolism, and proliferation in all eucaryote cells (Wang and Levine 2010; Musiwaro *et al.* 2013; Kim and Lee 2014; Birkenmeier *et al.* 2016; Allen and Baehrecke 2020). However, how autophagy organizes cellular signaling networks to exert these functions is not fully understood.

Here, we identified *atg1 ^+^* , a critical component of the autophagy machinery, as a gene that functions in the Pmk1 MAPK signaling pathway in fission yeast via a molecular genetic screening utilizing the *vic* ( v iable in the presence of i mmunosuppressant and c hloride ion) phenotype, which recapitulates Pmk1 MAPK signaling inhibition (Methods). The rationale of the *vic* mutant screening is based on the antagonistic relationship between Pmk1 MAPK and calcineurin, a highly conserved serine/threonine protein phosphatase, in the Cl ^−^ homeostasis in fission yeast. Knockout of the *S. pombe* calcineurin gene *ppb1* ^+^ or inhibition of calcineurin activity by the immunosuppressant FK506, a specific inhibitor of calcineurin, results in hypersensitivity to Cl ^−^ (Sugiura *et al.* 1998). This phenotype associated with *ppb1* deletion was reversed by the inhibition or gene knockout of the components of the Pmk1 MAPK signaling pathways, including Pmk1 MAPK, Pek1 MAPKK, Mkh1 MAPKKK, and Pck2 Protein kinase C (Sugiura *et al.* 1998, 1999; Ma *et al.* 2006). Our previous genetic screening to isolate *vic* mutants identified upstream activating regulators of MAPK signaling such as geranylgeranyl transferase (Doi *et al.* 2015) and farnesyl transferase (Ma *et al.* 2006).

The growth of the wild-type (WT) cells was significantly inhibited in the presence of the calcineurin inhibitor FK506 and 0.09 M MgCl _2_ , whereas knockout of the *pmk1* ^+^ gene makes cells grow much faster in the same condition (Figure 1A, Δ *pmk1* ). Δ *atg1* cells grew faster than the WT cells in the presence of FK506 and 0.09 M MgCl _2_ , although the growth of Δ *atg1* cells was slower than that of Δ *pmk1* (Figure 1A, Δ *atg1* ). The WT, Δ *pmk1 and* Δ *atg1* cells exhibited essentially the same pattern of sensitivity and resistance to FK506 and Cl ^−^ on EMM, YPD and YES plates, although the sensitivity of the WT cells and Δ *atg1* cells was more enhanced on YES than the other plates. Genetic interaction between Δ *atg1* and Δ *pmk1* was further examined by constructing Δ *atg1* Δ *pmk1* double mutant cells. The degree of the *vic* phenotype in Δ *atg1* Δ *pmk1* double mutants and Δ *pmk1* cells was almost equivalent. These results are consistent with Atg1 working upstream of the Pmk1 pathway. The difference in growth of these strains is almost indiscernible in the absence of FK506 and MgCl _2_ (Figure 1A).

To further explore the functional relationship between Atg1 and Pmk1 MAPK signaling, we utilized the cell growth inhibition associated with Pck2 overexpression. Pck2 overexpression in the WT cells leads to Pmk1 MAPK hyperactivation and cytotoxicity, which can be suppressed by the inhibition or knockout of the components of the Pmk1 MAPK pathway (Figure 1B) (Ma *et al.* 2006). This phenotypic evaluation also led to the identification of an SH3 adaptor protein Skb5 as a negative regulator of Pck2/Pmk1 signaling (Kanda *et al.* 2016). As shown in Figure 1B, *atg1* deletion significantly suppressed the toxicity induced by Pck2 overexpression, although the impact of *atg1* deletion on the suppression of the toxicity of Pck2 overexpression was smaller than that achieved by *pmk1* deletion. Thus, *atg1* deletion is likely to ameliorate Pck2-mediated Pmk1 MAPK hyperactivation.

Next, we confirm if the loss-of-function mutants of other autophagy-related genes also display the *vic* phenotype. As expected, a series of the deletion mutants of autophagy-related genes, except for *atg15 ^+^* , grew better than the wt strain in the media containing FK506 plus 0.09 M MgCl _2_ (Figure 1C). The degree of the *vic* phenotype of these *atg* mutants was similar to that of Δ *atg1* cells. These results suggest that the autophagy system as a whole may be involved in the Pmk1 MAPK signaling regulation.

In summary, our genetic screen revealed a functional interaction between autophagy-related genes and Pmk1 MAPK signaling in fission yeast. Several studies report functional crosstalk between MAPK signaling and autophagy, including the role of MAPK ERK in the maturation of autophagosomes (Corcelle *et al.* 2006), as well as the role of autophagosome as a scaffold to facilitate spatial coordination of RAF/MEK/ERK phosphorylation (Martinez-Lopez *et al.* 2013). Our epistasis analysis, showing the nonadditive *vic* phenotype of Δ *atg1* Δ *pmk1* double mutant and the resemblance of the Δ *atg1* Δ *pmk1* double mutant to the Δ *pmk1* single mutant, suggests that Atg1 acts upstream of the Pmk1 signaling pathway. Furthermore, suppressioin of the toxicity induced by Pck2 overexpression by *atg1* deletion suggests that Atg1 acts downstream of Pck2. MAPK signaling cascades consist of a core module of three tiers of protein kinases MAPK, MAPKK, and MAP3K, and often an additional upstream MAP4K. It would be intriguing if Atg1 serves as an additional layer of kinase mediating MAPK signaling activation. Future studies will elucidate the mechanism and the functional significance of the genetic interaction between autophagy and MAPK signaling revealed by our yeast genetic screen. Given the highly conserved nature of autophagy and MAPK signaling in the fate of cell death and proliferation, this study will provide valuable information to understand human diseases associated with aberrant regulation of MAPK signaling and autophagy.

## Methods

**Yeast strains, media, and molecular biology**

*Schizosaccharomyces pombe* strains and plasmids used in this study are listed in the Reagents section. The complete medium YPD and the minimal medium EMM have been described previously (Toda *et al.* 1996). FK506 was provided by Fujisawa Pharmaceutical Co. (Osaka, Japan). Standard genetic and recombinant-DNA methods (Moreno *et al.* 1991) were used except where noted.

***vic* mutant screening**

The growth of 89 viable kinase knockout strains generated by Bimbo *et al.* (Bimbó *et al.* 2005) was analyzed on the YPD plates with or without FK506 and 0.09 M MgCl _2_ by spotting growth assay.

**Spotting growth assay**

Yeast cells were cultured in 20 ml of liquid media at 27˚C till mid-log phase and diluted to 0.6 OD _660_ /ml, from which five 10-fold serial dilutions were prepared and then spotted onto the indicated plates. Plates were incubated at 27˚C for 3 to 7 days.

## Reagents

**Table:** 

**Strain**	**Genotype**			**Reference**
HM123	* h ^-^ leu1-32 *			Lab stock
KP2178	*h* ^-^ *leu1-32 pmk1* ::KanMX6			Lab stock
SP341	* h ^-^ leu1-32* *ura4-D18 atg1::ura4* ^+^			(Bimbó *et al.* 2009)
SP2857	* h ^-^ leu1-32* *ura4-D18 atg1::ura4* ^+^ *pmk1* ::KanMX6			This study
SK2	* h ^-^ leu1-32* *ura4-C190T atg2::ura4* ^+^			(Mukaiyama *et al.* 2009)
SK3	* h ^-^ leu1-32* *ura4-C190T atg3::ura4* ^+^			(Mukaiyama *et al.* 2009)
SK4	* h ^-^ leu1-32* *ura4-C190T atg4::ura4* ^+^			(Mukaiyama *et al.* 2009)
SK5	* h ^-^ leu1-32* *ura4-C190T atg5::ura4* ^+^			(Mukaiyama *et al.* 2009)
SK6	* h ^-^ leu1-32* *ura4-C190T atg6::ura4* ^+^			(Mukaiyama *et al.* 2009)
SK7	* h ^-^ leu1-32* *ura4-C190T atg7::ura4* ^+^			(Mukaiyama *et al.* 2009)
SK8	* h ^-^ leu1-32* *ura4-C190T atg8::ura4* ^+^			(Mukaiyama *et al.* 2009)
SK9	* h ^-^ leu1-32* *ura4-C190T atg9::ura4* ^+^			(Mukaiyama *et al.* 2009)
SK10	* h ^-^ leu1-32* *ura4-C190T atg12::ura4* ^+^			(Mukaiyama *et al.* 2009)
SK11	* h ^-^ leu1-32* *ura4-C190T atg13::ura4* ^+^			(Mukaiyama *et al.* 2009)
SK12	* h ^-^ leu1-32* *ura4-C190T atg15::ura4* ^+^			(Mukaiyama *et al.* 2009)
SK13	* h ^-^ leu1-32* *ura4-C190T atg17::ura4* ^+^			(Mukaiyama *et al.* 2009)
SK14	* h ^-^ leu1-32* *ura4-C190T atg22::ura4* ^+^			(Mukaiyama *et al.* 2009)

**Plasmid**	**Genotype**	**Promoter**	**Expressed protein**	**Reference**
pKB2728	pREP1-GFP	nmt1	GFP	Lab stock
pKB4763	pREP1-GFP-Pck2	nmt1	GFP-Pck2	(Ma *et al.* 2006)
